# The Relationship between Cardiovascular Risk Scores and Several Markers of Subclinical Atherosclerosis in an Asymptomatic Population

**DOI:** 10.3390/jcm10050955

**Published:** 2021-03-01

**Authors:** Ovidiu Mitu, Adrian Crisan, Simon Redwood, Ioan-Elian Cazacu-Davidescu, Ivona Mitu, Irina-Iuliana Costache, Viviana Onofrei, Radu-Stefan Miftode, Alexandru-Dan Costache, Cristian Mihai Stefan Haba, Florin Mitu

**Affiliations:** 1 1st Medical Department, Faculty of Medicine, University of Medicine and Pharmacy “Grigore T. Popa”, 700115 Iasi, Romania; mituovidiu@yahoo.co.uk (O.M.); ii.costache@yahoo.com (I.-I.C.); onofreiviana@gmail.com (V.O.); radu.miftode@yahoo.com (R.-S.M.); adcostache@yahoo.com (A.-D.C.); cristi.haba@gmail.com (C.M.S.H.); mitu.florin@yahoo.com (F.M.); 2 Department of Cardiology, Clinical Emergency Hospital “Sf. Spiridon”, 700111 Iasi, Romania; 3 Department of Cardiology, St. Thomas’ Hospital, Westminster Bridge Road, London SE1 7EH, UK; simon.redwood@gstt.nhs.uk; 4 Department of Cardiology, Clinical Emergency Hospital, 8 Calea Floreasca, 014475 Bucharest, Romania; elian.cazacu@gmail.com; 5 Department of Morpho-Functional Sciences II, Faculty of Medicine, University of Medicine and Pharmacy “Grigore T. Popa”, 700115 Iasi, Romania; 6 Department of Cardiovascular Rehabilitation, Clinical Hospital of Rehabilitation, 700661 Iasi, Romania

**Keywords:** subclinical atherosclerosis, SCORE, Framingham, QRISK, PROCAM, cardiovascular risk, pulse wave velocity, intima media thickness

## Abstract

Background: The current cardiovascular disease (CVD) primary prevention guidelines prioritize risk stratification by using clinical risk scores. However, subclinical atherosclerosis may rest long term undetected. This study aimed to evaluate multiple subclinical atherosclerosis parameters in relation to several CV risk scores in asymptomatic individuals. Methods: A cross-sectional, single-center study included 120 asymptomatic CVD subjects. Four CVD risk scores were computed: SCORE, Framingham, QRISK, and PROCAM. Subclinical atherosclerosis has been determined by carotid intima-media thickness (cIMT), pulse wave velocity (PWV), aortic and brachial augmentation indexes (AIXAo, respectively AIXbr), aortic systolic blood pressure (SBPao), and ankle-brachial index (ABI). Results: The mean age was 52.01 ± 10.73 years. For cIMT—SCORE was more sensitive; for PWV—Framingham score was more sensitive; for AIXbr—QRISK and PROCAM were more sensitive while for AIXao—QRISK presented better results. As for SBPao—SCORE presented more sensitive results. However, ABI did not correlate with any CVD risk score. Conclusions: All four CV risk scores are associated with markers of subclinical atherosclerosis in asymptomatic population, except for ABI, with specific particularities for each CVD risk score. Moreover, we propose specific cut-off values of CV risk scores that may indicate the need for subclinical atherosclerosis assessment.

## 1. Introduction

The current primary prevention guidelines for the management of cardiovascular diseases (CVD) prioritize risk identification, mostly through traditional CV risk factors and risk stratification by using clinical and risk scores [1,2,3,4,5]. Researchers have developed and validated multivariable risk prediction tools that synthesize CV risk-factor information to predict future CV events in different populations [5,6]. As CVD present a long asymptomatic phase, there has been support for the expansion of predictive studies of arterial disease from its clinical form to subclinical manifestation [7]. Several CVD predictive clinical tools have been developed, the most used being the SCORE risk for European countries [1], the Framingham risk score for USA [8], and the QRISK score for UK [9]. Nonetheless, the detection of subclinical atherosclerosis has been shown to be a useful method for predicting future CV events [10,11].

Atherosclerosis is widely recognized as a major cause of death and disability worldwide [12]. It manifests as a continuum from subclinical phase to patent clinical atherosclerotic CVD that starts early in life and remain clinically undetected until an acute CV event occurs [13,14]. Subclinical atherosclerosis is an early indicator of atherosclerotic burden and its timely recognition can slow or prevent the progression to overt CVD [15]. Thus, individuals with subclinical atherosclerosis require primary CVD prevention and, simultaneously, they represent a challenge in primary care setting. Further on, most people are at high risk for acute CV events but are not aware because their traditional risk-factor levels are not unusually high [16]. However, the current data is rather limited regarding the value of CV risk scores associated with the presence of subclinical atherosclerosis, especially in asymptomatic populations.

Thus, our study aimed to: (i) evaluate subclinical atherosclerosis in asymptomatic individuals using multiple risk prediction scores, (ii) establish cut-off values of the CVD risk scores in predicting the presence of subclinical atherosclerosis markers, and (iii) determine the variance and unit modifications of CVD risk scores in relationship to subclinical atherosclerosis parameters.

## 2. Materials and Methods

### 2.1. Study Design and Population

A cross-sectional, single-center, observational study was conducted over a two-year period and aimed to evaluate only asymptomatic CVD subjects. The “asymptomatic” status was defined as having no previous recordings of any acute or chronic diseases and not being under any chronic medical treatment. As inclusion criteria, only apparently healthy subjects, aged 35–75 years, were proposed for evaluation from the general practitioners’ (GP) data lists. Moreover, pregnant or breastfeeding women were not eligible, as well as persons that refused or could not consent the study participation. Initially, 703 subjects were randomized from the GP data lists, resulting in 276 apparently healthy individuals that met the inclusion criteria. Further eligibility was ascertained by telephone interview, part of them refused or did not present to the study visit, resulting in 120 subjects that were finally evaluated.

The study protocol had been approved by the local Ethics Committee and all participants signed an informed written consent before enrolment. The protocol and the used methods adhered to the Helsinki Declaration.

At the study visit, a detailed medical interview was conducted in all participants. The traditional CV risk factors were analyzed along with a complete physical examination that assessed the anthropometric parameters as well. The office blood pressure was measured according to the ESC/ESH guidelines recommendations [17]. A fasting venous blood sample was collected for biochemical analysis that included lipid profile, plasma glucose, and renal and hepatic function.

### 2.2. CVD Risk Scores

Based on the CV risk factors obtained from the medical interview, physical examination and biochemical tests, four major CV risk scores were computed: HeartScore^®^, Framingham, QRISK^®^3, and PROCAM. HeartScore^®^ was developed by applying the SCORE (Systematic Coronary Risk Evaluation Project) risk and derived from European population. We aimed to use different risk scores that were validated on different populations for a comprehensive overview. For a uniform analysis, all scores predict the 10-year CVD risk, but every score is based on different risk factors, the most facile being HeartScore^®^ while QRISK^®^3 presents the most exhaustive evaluation [1,8,9,18]. Table 1 summarizes the main characteristics of all four CV risk scores.

### 2.3. Subclinical Atherosclerosis Evaluation

The subclinical atherosclerosis was evaluated by several standardized methods: carotid ultrasound for carotid intima-media thickness (cIMT), arterial stiffness parameters, and ankle-brachial index (ABI).

cIMT and carotid plaques were assessed by using ultrasonography and performed by a physician blinded to all patient data and respecting the Mannheim criteria [19]. A cIMT value > 0.9 mm was considered abnormal.

Arterial stiffness was evaluated using an Arteriograph^®^ system device which uses the oscillometric method for determination. Its results have been validated in previous studies [20,21]. Besides pulse wave velocity (PWV), the device also provided other arterial stiffness markers such as aortic and brachial augmentation indexes (AIXao, respectively AIXbr) or aortic systolic blood pressure (SBPao).

ABI was performed in a standardized method by a single trained operator. A ratio < 0.9 was considered pathological for defining peripheral artery disease. The lowest value from either leg was introduced into the final analysis.

### 2.4. Statistical Analysis

Statistical analysis was performed in IBM SPSS Statistics 22.0, US. The existence of a relationship between variables was evaluated by Pearson correlation coefficient and a linear regression equation was conducted to observe how two or more variables vary between them. To establish the most appropriate cut-off values of the CV risk scores in predicting the presence of subclinical atherosclerosis markers, ROC curves and the area under the curve (AUC) were used for the benefit of using the test(s) in question. Descriptive data is presented as mean ± standard deviation. A *p*-value < 0.05 was considered statistically significant.

## 3. Results

The clinical and biological characteristics of the study group are highlighted in Table 2. The mean age of patients was 52.01 ± 10.73 years, with one third being males and all of them had Caucasian ethnicity. Among traditional risk factors, more than 20% were smokers, 30% presented positive family history of CVD, mean body mass index (BMI) was 28 kg/m^2^. Average blood pressure (BP) was in normal ranges, however, 28.3% of them had undiagnosed arterial hypertension. Mean lipid parameters were at the superior borderline limit while the plasma glucose level and renal function were mostly normal. Regarding subclinical atherosclerosis, about 40% of the subjects presented carotid ultrasound abnormalities, the majority had normal ABI, while 20% showed increased arterial stiffness parameters. Overall, average CV risk scores included population at intermediate risk.

In univariate analysis, all four CV risk correlated significantly with cIMT, PWV, SBPao, AIXao, AIXbr (*p* < 0.05). As well, the presence of carotid plaques was associated with increased CV risk scores (*p* < 0.001). However, ABI alterations were not associated with increased CV risk scores.

**Figure 1:**
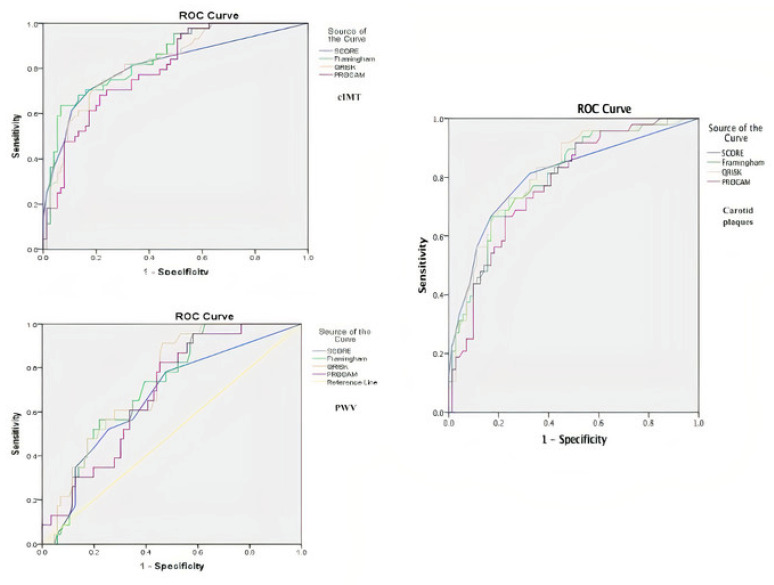
ROC curves of CV risk scores in association with subclinical atherosclerosis parameters (cIMT, carotid intima-media thickness; PWV, pulse wave velocity)

Moreover, for each major determinant of subclinical atherosclerosis (cIMT, carotid plaques, PWV), the CV risk scores were introduced into the ROC curves (Figure 1), obtaining values with (AUC) more than 0.600 so we can consider our ROC curve significantly better than chance and relevant for a diagnostic. For cIMT, Framingham was the best score associated with increased values of this parameter, closely followed by SCORE and QRISK (Table 3). The same three scores presented rather similar values in predicting the presence of carotid plaques (Table 4). As for PWV, the AUC were a bit smaller, with QRISK and Framingham having the best performances (Table 5). Nonetheless, all CV risk scores significantly predicted the presence of subclinical atherosclerosis irrespective of the used method (*p* < 0.05).

Going into further analysis, we tried to obtain several cut-off points for CV risk scores that could predict the presence of subclinical atherosclerosis (Table 6). To investigate the cut-off points, we had to balance the importance of the false positive rate (FPR) in our analysis. Since we aimed the diagnosis for the presence of subclinical atherosclerosis, the repercussions for the FPR will only address the preventive concerns. That could only help the patient in order to prevent the development of subclinical atherosclerosis even though the patient is at low or intermediate CV risk. Thus, a higher FPR can be considered as necessary so that the result will not harm the patient under any circumstances. Assuming this hypothesis for identifying the most significant cut-off values that may suggest the screening for subclinical atherosclerosis, we considered that we could accept an increased value of FPR as long as the sensitivity is higher than 0.700, mostly 0.800, but with as lower specificity as possible. Nonetheless, for higher sensibility, we were willing to accept a moderate percentage of patients with FPR because the indicated tests are not invasive and widely available, and the subclinical atherosclerosis assessment could bring valuable information.

Thus, a SCORE > 1.5 could indicate the presence of subclinical atherosclerosis regardless of the used method. Framingham values > 7.95 could indicate the presence of carotid modifications, while higher values are needed for an increase in PWV. For QRISK, the values are rather irregular, nonetheless values > 5.3 may represent a sign of subclinical atherosclerosis, while PROCAM values > 2.1 may trigger the attention towards subclinical changes.

By performing multivariate regression analysis for the relationship between different parameters of subclinical atherosclerosis and CV risks scores, a statistically significant correlation was obtained for all four CV risk scores. Thus, a regression equation was performed in order to predict the change in every risk score for detecting subclinical atherosclerosis parameters in the study group (Table 7). We interpret the results, from the point of view of sensitivity, as follows:

- For cIMT—SCORE is more sensitive (33% of the variance in cIMT was predictable from SCORE); each increase of SCORE with 1.16 signifies a further increase of cIMT with 0.1 mm. The prediction was closely followed by Framingham (29% variance) and QRISK (28% variance).
- For PWV—Framingham score is more sensitive (21% of the variance in PWV was predictable from Framingham score). This result can be translated that for each increase of Framingham value with 2.1, PWV increases as well with 1 m/s. The prediction was closely followed by QRISK (19% variance) and SCORE (17% variance).
- For AIXbr—QRISK and PROCAM are more sensitive, but all risk scores present a variance <10%.
- For AIXao—QRISK is more sensitive, but all risk scores present a variance <10%.
- For SBPao—SCORE is more sensitive (23% of the variance of SBPao was predictable from SCORE); each increase of SCORE with 0.6 signifies a further increase of SBPao with 10 mmHg. The prediction was closely followed by Framingham (21% variance) and QRISK (18% variance).
- For ABI—PROCAM score is more sensitive, but the overall prediction values are ≤0.1%.

## 4. Discussion

The current guidelines for primary CVD prevention recommend initial assessment and risk stratification based on traditional risk factor scoring followed by therapeutic intervention when necessary [1,6,22,23,24,25]. However, risk scores have been developed to predict the risk of clinical evident CVD rather than subclinical changes. By comparing to other studies, our findings add novel data to the relationship between current CV risk evaluation based on risk scores and subclinical atherosclerotic evidence. It represents one of the fewest studies that correlated several risk scores with different markers of subclinical atherosclerosis, proposing specific cut-off values that would require a comprehensive CV evaluation in asymptomatic population.

Subclinical atherosclerosis parameters have proven their utility in clinical practice, both in primary and secondary CV prevention. In asymptomatic populations, increased values of coronary artery calcium score determined by computed tomography, several arterial stiffness markers, ABI or peripheral arterial modifications (carotid, aortic or iliofemoral) determined by ultrasound have been highly prevalent and detected as well in intermediate and low-risk subjects, not only in those with already high computed CV risk [26,27]. Moreover, increased subclinical atherosclerosis parameters have been correlated with CV risk events on long term.

According to current recommendations, the presence of carotid plaque is viewed as a high risk finding (≥10% cardiovascular mortality risk at 10 years) [1]. In our study, all cardiovascular risk scores increased, in a directly proportional way, in patients with carotid plaques. Even if the sensibility was related to all four risk scores, the specificity was better for SCORE. These finding were similar with a study conducted by Romanens Michael et al., on 3.248 patients, aged 40–65, with no medication and no CV risk factors. The authors assessed the prevalence of “old” arteries (vascular age ≥70 years) using carotid plaque thickness. The results showed that most subjects with “old” arteries were classified as low risk according to PROCAM, while for SCORE only 20% of patients were in the low-risk group. Both scores correlated with carotid plaques, but the specificity and sensibility were better for SCORE [23]. Some studies assumed that the Framingham risk score underestimated subclinical atherosclerosis risk in asymptomatic individuals. In a carotid ultrasonography study, the echography assessment of subclinical CVD improved the reclassification of one-third of subjects with low or intermediate Framingham score into higher risk groups [24]. Rather similar, in a study on 662 patients without known CVD, 33.8% of patients who had been classified as low risk by the Framingham risk score presented subclinical coronary artery atherosclerosis detected by electron beam computed tomography. Despite this, they did not meet the criteria for pharmacologic therapy as defined by the score [25]. Another study aimed to investigate the features of subclinical carotid plaques in 166 asymptomatic patients with at least one CVD risk factor, by using multi-contrast weighted MRI and to correlate these findings with Framingham risk score. Sixty-six percent of the intraplaque hemorrhage occurred in low and intermediate-risk groups according to Framingham stratification. Therefore, Framingham risk score was not specific for carotid plaque because the stratification failed to identify more than half of individuals with complicated carotid plaque. Furthermore, nearly 1/3 of the individuals in the low-risk Framingham stratification had lipid rich necrotic core at the carotid assessment [6]. In our study all CV risk scores showed a directly proportional increase in patients with carotid plaques, but the specificity was superior for SCORE.

Carotid IMT is a strong predictor of CV events independent of conventional risk factors [28]. Juho RH Raiko et al. examined the carotid modifications in 2204 patients, aged 24–29 years, who were followed for 6 years. The authors used Framingham, SCORE, and PROCAM risk scores to predict subclinical atherosclerosis. All risk scores had equal performance in the prediction of 6-year increased cIMT and carotid plaques (*p* < 0.05) [29]. Another cohort study that followed 1348 subjects (18–99 years) over 12.7 years showed that 115 subjects developed nonfatal ischemic stroke, transient ischemic attack or vascular death. The inclusion of carotid findings (presence of cIMT > 1 mm or present plaque) resulted in a higher predictive power than Framingham risk score alone among those with a score > 20% [30,31]. In an observational, cross-sectional cohort study on 362 hypertensive subjects, cIMT correlated positively with the CV risk estimated by both SCORE (*r* = 0.421, *p* < 0.01) and Framingham (*r* = 0.363, *p* < 0.01) [32]. There was a significant positive correlation between cIMT and the Framingham risk score (*r* for men = 0.571; *p* < 0.001; *r* for women = 0.633; *p* < 0.001) and there was no significant gender difference between these two groups [33]. Furthermore, in a recent meta-analysis of 119 clinical trials involving 100.667 patients, the interventions reducing cIMT progression by 10, 20, 30, or 40 μm/year would yield CV relative risks of 0.84 (0.75–0.93), 0.76 (0.67–0.85), 0.69 (0.59–0.79), or 0.63 (0.52–0.74). In conclusion, the extent of interventions effects on cIMT progression predicted the degree of CV risk reduction [34]. We obtained similar results in our study, suggesting that the additional risk factors included in PROCAM (parental history of myocardial infarction and regional adjustment factor based on geographic prevalence) did not increase discrimination in our cohort. Moreover, although lacking HDL-cholesterol and diabetes status, SCORE showed an equal value in predicting high cIMT.

Regarding ABI evaluation, we did not find any associations with increased CV risk scores. A study conducted on 6091 patients aged ≥40 years, without any CVD, aimed to assess a link between subclinical atherosclerosis (determined by ABI) and different CV risk scores. Compared to individuals classified as low-risk by Framingham, individuals at intermediate-risk were not prone to have subclinical atherosclerosis, though individuals classified at high-risk had a two-fold increase of subclinical atherosclerosis (OR 2.31; 95%CI: 1.53–3.49). As for the sensitivity and specificity analysis, high-risk patients (vs. low-risk) had the lowest sensitivity (26.6%) and most specificity (87.4%) for identifying subclinical atherosclerosis. Intermediate-risk patients (vs. low-risk) had slightly better sensitivity (33.9%), but also lower specificity (64.9%) [5]. In a cross-sectional study on 6292 patients aged ≥40 years, without known CVD or diabetes, there was a close relation between abnormal ABI and Framingham risk score. 91.4% of patients were at < 20% Framingham risk score, and, out of these, only 2.7% (95%CI: 2.3–3.1%, *p* < 0.0001) had an abnormal ABI. The results showed that abnormal ABI is highly prevalent among individuals at low-intermediate Framingham risk score [35]. However, our results are divergent regarding the ABI screening possibly due to the sample size and the population characteristics.

As for PWV and CV risk scores, all four CV risk scores correlated significantly with PWV in our study. Similar results were obtained by L. Woźnicka-Leśkiewicz et al. where 200 patients were randomized into four different groups. In the group characterized by the lack of CV risk factors, PWV correlated significantly with the CV risk according to SCORE scale (*r* = 0.45, *p* < 0.001) and Framingham risk score (*r* = 0.37, *p* < 0.001) [36]. In a prospective study conducted on 177 subjects without evidence of significant CVD, the authors assessed the association between carotid augmentation index (CAI), carotid femoral PWV (cfPWV) and Framingham risk score. There was a significant association between cfPWV and Framingham score (*r* = 0.417, *p* < 0.001) and a weaker but also significant relation between CAI and Framingham score (*r* = 0.267, *p* < 0.001). cfPWV was significantly related to Framingham score in both men and women (*p* < 0.001 in both sexes), whereas the relation between CAI and Framingham score was significantly only in women (*p* < 0.001). The study suggested that cfPWV may be associated with CVD risk irrespective of sex, whereas CAI may be associated with CVD risk in women only [37]. Our study supports these results in terms of PWV (Framingham score was more sensitive) but not for AIX where QRISK and PROCAM were more sensitive, but all risk scores presented a variance <10%. Another study obtained slightly different results from our research, conducted with the aim to evaluate the association between SCORE risk and cfPWV, with a follow up of 4.9 years. A strong association between high CVD risk (SCORE ≥ 5%) and high PWV (OR 2.29; 95%CI 1.17–4.46) has been obtained [38].

Regarding AIXbr or AIXAo in our study, QRISK was the most sensitive. In another cross-sectional study on 81 young and middle-aged males (39.2 ± 6.3 years) without evidence of overt CVD or diabetes, the Framingham risk score was significantly correlated with AIXAo (*r* = 0.266, *p* = 0.009) [39]. Moreover, increased AIXAo was associated with a high Framingham risk score in patients that were referred to percutaneous coronary intervention (PCI) compared to non-PCI group (AIX was analyzed before coronary angiography) [40].

As CV risk scores are associated with subclinical atherosclerosis parameters, we defined specific cut-off values that may impose the screening for subclinical atherosclerosis. Thus, based on the results summarized in Table 6 and combined with the pre-defined risk categories for each CV score but taking into consideration the need for not abusing these methods, we suggest the screening of subclinical atherosclerosis in subjects with SCORE ≥ 3, Framingham ≥ 10, QRISK ≥ 10 or PROCAM ≥ 5, irrespective of used method.

Our study may present some limitations. Firstly, the moderate sample size population could represent a limiting factor for the divergent results obtained for ABI. As well, others novel markers of subclinical atherosclerosis could have been implemented as the coronary calcium score. However, we have used non-invasive methods that are easy for use in clinical practice and, added to the CV scores, could better refine the CV risk.

## 5. Conclusions

In the current study, all four CV risk scores were associated with markers of subclinical atherosclerosis in an asymptomatic population, except for ABI. The SCORE risk was better associated with carotid ultrasound abnormalities while Framingham and QRISK seemed more specific for increased arterial stiffness parameters. Moreover, we proposed specific cut-off values of CV risk scores that may indicate the need for subclinical atherosclerosis assessment. However, further research is needed for a tailored CV risk refinement based on risk scores and subclinical atherosclerosis markers.

## Figures and Tables

**Table 1:** Main characteristics of the cardiovascular disease (CVD) risk scores.

	HeartScore^®^	Framingham	QRISK^®^3	PROCAM
Population assessed	European countries	USA	UK	Germany
Time prediction	10 years	10 years	10 years	10 years
Outcomes	Fatal CVD	Fatal and non-fatal CVD	Incident CVD	Fatal and non-fatal CVD
Number of risk factors	5	8	20 (8—non CVD major risk factors)	9
Year of last developed model	2003	2008	2017	2007

**Table 2:** General characteristics of the study population.

General Characteristics	Specific Characteristics		All Subjects (*n* = 120)
Risk factors	Age, years		52.01 ± 10.73
	Male, *n* (%)		40 (33.3)
	Smoking status	Current smoker, *n* (%)	26 (21.6)
		Former smoker, *n* (%)	22 (18.3)
		Never smoker, *n* (%)	72 (60)
	Alcohol consumers, *n* (%)		15 (12.5)
	Family history of CVD *, *n* (%)		36 (30)
	Body mass index, kg/m²		28.50 ± 5.34
	Waist circumference, male, cm		103.60 ± 10.29
	Waist circumference, female, cm		97.2 ± 13.62
	Systolic blood pressure, mmHg		127.30 ± 17.22
	Diastolic blood pressure, mmHg		81.27 ± 13.07
	Cholesterol total, mg/dL		209.77 ± 45.56
	LDL cholesterol, mg/dL		129.96 ± 40.71
	HDL cholesterol, mg/dL		52.49 ± 14.47
	Non-HDL cholesterol, mg/dL		157.27 ± 44.89
	Triglycerides, mg/dL		137.06 ± 81.42
	Plasma glucose, mg/dL		97.21 ± 12.75
	eGFR, ml/min/1.73 m²		89.35 ± 16.54
Subclinical atherosclerosis	cIMT, mm		0.86 ± 0.13
	cIMT > 0.9 mm, *n* (%)		44 (36.7)
	Carotid plaques, *n* (%)		48 (40)
	ABI		1.08 ± 0.13
	PWV, m/s		8.28 ± 1.79
	PWV > 10 m/s, *n* (%)		23 (20.9)
	Aortic systolic blood pressure, mmHg		128.14 ± 21.05
	AIXbr, %		−0.98 ± 31.03
	AIXao, %		37.04 ± 15.60
CVD risk charts	SCORE risk		2.95 ± 2.71
	Framingham		10.29 ± 8.38
	QRISK		7.23 ± 6.93
	PROCAM		5.49 ± 7.83

ABI indicates ankle-brachial index; AIXao, aortic augmentation index; AIXbr, brachial augmentation index; cIMT, carotid intima-media thickness; CVD, cardiovascular diseases; eGFR, estimated glomerular filtration rate; HDL, high-density lipoprotein; LDL, low-density lipoproteins; PWV, pulse wave velocity; * acute atherosclerotic events for men <55 years of age and women <65 years of age in first degree relatives.

**Table 3:** Relationship between cIMT and CVD risk scores (by ROC analysis).

Test Result Variable(s)	Area	Std. Error	Asymptotic Sig.	Asymptotic 95% Confidence Interval	
				Lower Bound	Upper Bound
SCORE	0.812	0.044	0.000	0.726	0.897
Framingham	0.845	0.036	0.000	0.774	0.916
QRISK	0.825	0.038	0.000	0.749	0.900
PROCAM	0.796	0.040	0.000	0.717	0.875

**Table 4:** Relationship between carotid plaques and CVD risk scores (by ROC analysis).

Test Result Variable(s)	Area	Std. Error	Asymptotic Sig.	Asymptotic 95% Confidence Interval	
				Lower Bound	Upper Bound
SCORE	0.803	0.043	0.000	0.719	0.887
Framingham	0.799	0.041	0.000	0.720	0.879
QRISK	0.811	0.040	0.000	0.733	0.889
PROCAM	0.774	0.043	0.000	0.690	0.857

**Table 5:** Relationship between PWV and CVD risk scores (by ROC analysis).

Test Result Variable(s)	Area	Std. Error	Asymptotic Sig.	Asymptotic 95% Confidence Interval	
				Lower Bound	Upper Bound
SCORE	0.666	0.062	0.015	0.545	0.787
Framingham	0.715	0.052	0.002	0.613	0.817
QRISK	0.733	0.050	0.001	0.635	0.832
PROCAM	0.688	0.054	0.006	0.582	0.794

**Table 6:** Cut-off values of the CVD risk scores in predicting the presence of subclinical atherosclerosis markers.

Risk Score		cIMT	PWV	Carotid Plaques
SCORE	Cut-off value	1.5	1.5	1.5
	Sensitivity (%)	81.8	78.3	81.3
	Specificity (%)	64.5	51.7	66.7
	PPV (%)	57.1	30	61.9
	NPV (%)	86	90	84.2
Framingham	Cut-off value	7.95	8.8	4.75
	Sensitivity (%)	81.8	73.9	89.6
	Specificity (%)	65.8	59.8	51.4
	PPV (%)	58.1	32.7	55.1
	NPV (%)	86.2	89.7	88.1
QRISK	Cut-off value	5.3	3.7	4.3
	Sensitivity (%)	81.8	95.7	83.3
	Specificity (%)	68.4	46	63.9
	PPV (%)	60	31.9	60.6
	NPV (%)	86.7	97.6	85.2
PROCAM	Cut-off value	2.11	2.05	1.62
	Sensitivity (%)	77.3	82.6	81.2
	Specificity (%)	63.2	54	58.3
	PPV (%)	54.8	32.2	56.5
	NPV (%)	82.8	92.2	82.4

cIMT indicates carotid intima-media thickness; NPV, negative predictive value; PPV, positive predictive value; PWV, pulse wave velocity.

**Table 7:** Variance and unit modifications of CVD risk scores in relationship to subclinical atherosclerosis parameters.

	Linear Regression	PWV (Increase with One Unit)	cIMT Max (Increase with 0.1)	ABI (Increase with 0.1)	AIXbr (Increase with 1%)	AIXao (Increase with 1%)	SBPao (Increase with 1 mmHg)
SCORE	*r*	0.41	0.57	−0.10	0.27	0.28	0.48
	*p*	<0.01	<0.01	<0.01	0.01	<0.01	<0.01
	Increase/decrease	0.6	1.16	0.2	0.025	0.05	0.06
	R^2^	0.17	0.33	0.01	0.07	0.07	0.23
	Reg. ec.	y = −2.27 + 0.65 *x	y = −6.96 + 11.46 *x	Y = 5.32 − 2.19 *x	Y = 3.11 + 0.02 *x	Y = 1.23 + 0.05 *x	Y = −5.06 + 0.06 *x
Framingham	*r*	0.45	0.54	−0.11	0.25	0.25	0.46
	*p*	<0.01	<0.01	<0.01	<0.01	<0.01	<0.01
	Increase/decrease	2.1	3.3	0.6	0.069	0.14	0.18
	R^2^	0.21	0.29	0.01	0.06	0.06	0.21
	Reg. ec.	y = −7.49 + 2.19 *x	y = −18.64 + 33.48 *x	Y = 17.71 − 6.86 *x	Y = 10.71 + 0.07 *x	Y = 5.46 + 0.14 *x	Y = −13.47 + 0.19 *x
QRISK3	*r*	0.44	0.53	−0.07	0.27	0.28	0.42
	*p*	<0.01	<0.01	0.04	<0.01	<0.01	<0.01
	Increase/decrease	1.75	2.7	0.3	0.063	0.12	0.14
	R^2^	0.19	0.28	0.005	0.07	0.08	0.18
	Reg. ec.	y = −7.06 + 1.76 *x	y = −16.11 + 27.01 *x	Y = 11.08 − 3.55 *x	Y = 2.75 + 0.13 *x	Y = 2.75 + 0.13 *x	Y = −10.84 + 0.14 *x
PROCAM	*r*	0.37	0.42	−0.15	0.27	0.28	0.29
	*p*	<0.01	<0.01	0.04	<0.01	<0.01	<0.01
	Increase/decrease	1.68	2.4	0.8	0.073	0.14	0.11
	R^2^	0.14	0.18	0.02	0.07	0.07	0.08
	Reg. ec.	y = −8.2 + 1.68 *x	y = −15.56 + 24.32 *x	Y = 14.92 − 8.71 *x	Y = 0.22 + 0.15 *x	Y = 0.22 + 0.15 *x	Y = −8.6 + 0.11 *x

ABI indicates ankle-brachial index; AIXao, aortic augmentation index; AIXbr, brachial augmentation index; cIMT, carotid intima-media thickness; PWV, pulse wave velocity; SBPao, aortic systolic blood pressure; Reg. ec. = regression equation.

## Data Availability

The data presented in this study are available on request from the corresponding author.
